# Deconstructing the nature of emotion regulation impairments at the identification, selection, and implementation stages in individuals at clinical high-risk for psychosis

**DOI:** 10.1017/S0033291724003155

**Published:** 2025-02-05

**Authors:** Gregory P. Strauss, Ian Raugh, Katherine Visser, Elaine Walker, Vijay Mittal

**Affiliations:** 1 Department of Psychology, University of Georgia; 2 Department of Psychiatry and Human Behavior, The Warren Alpert Medical School, Brown University; 3 Department of Psychology, Emory University; 4 Department of Psychology, Northwestern University

**Keywords:** Affect, Digital phenotyping, Stress, Extended process model

## Abstract

**Background:**

Psychotic disorders are characterized by emotion regulation abnormalities that predict greater symptom severity and poor functional outcomes. However, it is unclear whether these abnormalities also occur in individuals at clinically high risk for psychosis (CHR). The current study used ecological momentary assessment (EMA) to address this question and examined the nature of abnormalities at three stages of emotion regulation (identification, selection, implementation).

**Methods:**

Participants included 120 CHR and 59 CN who completed 1 week of EMA surveys evaluating emotional experience, emotion regulation, context, and symptoms. Multi-level models examined concurrent and time-lagged effects.

**Results:**

CHR evidenced elevated state negative affect and abnormalities at all three stages of emotion regulation. At the identification stage (i.e., determining the need to regulate), regulatory attempts were made too frequently and with too much effort at low levels of negative affect and not frequently enough and with insufficient effort at high levels of negative affect. Selection stage abnormalities (i.e., choosing the exact strategy to attempt based on context) were characterized by increased frequency of selecting individual strategies and greater polyregulation (i.e., use of multiple strategies concurrently). Implementation stage (i.e., executing the selected strategy) abnormalities were indicated by being less effective at decreasing the intensity of negative affect from time t to t + 1.

**Conclusions:**

It is not only heightened stress reactivity that confers risk for psychosis, but also abnormalities in applying emotion regulation strategies to control the stress response. The profile of abnormalities observed in CHR is similar to schizophrenia, suggesting treatment targets that transcend phases of psychotic illness.

## Introduction

Psychotic disorders are associated with substantial suffering and are a leading cause of medical disability in the United States (Rössler, Salize, Van Os, & Riecher-Rössler, 2005; Salomon et al., 2013). Given that few individuals achieve recovery after illness onset (Harrow & Jobe, 2007), there has been increasing interest in the early identification and prevention of psychosis (Fusar-Poli et al., 2013). Psychotic disorders are typically preceded by a prodromal (i.e., pre-illness) phase characterized by functional decline and subthreshold positive symptoms that progressively worsen over the course of several months to years (Fusar-Poli et al., 2013). It is now possible to reliably identify a group of clinical high-risk (CHR) youth who will go on to develop a psychotic disorder using state-of-the-art clinical interviews that evaluate attenuated positive symptom criteria. However, the majority of youth (~75%) who are identified as CHR do not develop a psychotic disorder within two years and it remains unclear which pathophysiological processes are most predictive of psychosis risk (Fusar-Poli et al., 2012).

The stress-vulnerability model remains one of the leading theories on the origins of psychosis (Walker & Diforio, 1997). This model proposes that dysfunction within the hypothalamic–pituitary–adrenal (HPA) axis reflects a latent vulnerability that triggers a cascade of neural processes that culminate in psychosis when environmental stressors are experienced (Walker, Mittal, & Tessner, 2008a). Although HPA axis dysfunction alone predicts conversion to psychosis among CHR youth (Walker et al., 2010), interventions designed to augment this mechanism have not led to breakthroughs in prevention. As such, there is a critical need to develop novel targets for early intervention and evaluate alternative vulnerability processes involved with psychosis risk and probability of conversion.

One means of addressing this need is by identifying mechanisms underlying emotion regulation (ER) impairments (i.e., the use of strategies to control the frequency, duration, or intensity of emotional response) (Gross, 1998). Although several theoretical models of ER have been proposed, the *Extended Process Model* (Gross, 2015) offered by James Gross has received the most empirical attention. Gross (Gross, 2015) posits that separate, but interactive systems for emotion generation and regulation exist. Both of these systems unfold over a cycle that involves four components: (1) World (W), which consists of internal and external stimuli that give rise to Valuation; (2) Perception (P), which includes the processing of personally salient emotional stimuli from the environment or internally generated mental representations that are gated into working memory where they can then be subjected to more elaborative processing; (3) Valuation (V), which involves determination of whether W is good or bad for the individual based on a cost–benefit analysis of past experiences and the current context. This stage also evaluates the discrepancy between the current state and the goal state generated in W (e.g., I feel angry now; my goal is to not feel angry). If the discrepancy is determined to be above a critical threshold, then the cycle will identify a target goal (e.g., reducing anger) and proceed to the next step where the goal can be acted upon; (4) Action (A) involves the initiation of a response to reduce the discrepancy identified in the V stage. Once A is complete and a new W is created, the emotion generation sequence may restart. However, when the first W, P, V, A emotion generation cycle identifies a goal to change the current emotional state, ER processes are then initiated in the form of a second-order W, P, V, A cycle. When engaged, the second-order system can activate one of five ER strategies (situation selection, situation modification, attentional deployment, reappraisal, expressive suppression). These strategies are completed following three sequential ER processes: identification (i.e., after an emotion is detected, determining whether to regulate or not), selection (i.e., choosing a contextually appropriate strategy), and implementation (i.e., executing the strategy that has been selected) (Sheppes, Suri, & Gross, 2015).

Although several etiological models emphasize the role of heightened stress reactivity in the emergence and maintenance of schizophrenia (SZ) (Walker et al., 2010; Walker & Diforio, 1997; Walker, Mittal, & Tessner, 2008a; Walker, Mittal, & Tessner, 2008b), relatively few studies have evaluated ER in the schizophrenia spectrum and no study has systematically evaluated whether abnormalities occur at the three stages of Gross’ model (identification, selection, implementation) in CHR youth. To date, most published studies have evaluated self-reported ER strategy use via questionnaires, generally finding that SZ patients and CHR youth self-report less frequent use of reappraisal and greater use of suppression than healthy controls (CN) (Chapman et al., 2020; Horan, Hajcak, Wynn, & Green, 2013a; Kimhy et al., 2012; Kimhy et al., 2016; Livingstone, Harper, & Gillanders, 2009; Perry, Henry, & Grisham, 2011; Rowland et al., 2013; van der Meer, van’t, & Aleman, 2009). Additionally, lower use of reappraisal (an adaptive strategy) and greater use of suppression (a maladaptive strategy) predicts a range of poor clinical outcomes, including positive symptoms, negative symptoms, and social functioning (Chapman et al., 2020; Horan, Hajcak, Wynn, & Green, 2013a; Kimhy et al., 2012; Kimhy et al., 2016; Livingstone, Harper, & Gillanders, 2009; Perry, Henry, & Grisham, 2011; Rowland et al., 2013; van der Meer, van’t, & Aleman, 2009). These questionnaire studies provide important evidence that an ER abnormality exists in SZ and CHR youth; however, they do not provide an indication of *why* this deficit occurs and which stages of ER are abnormal.

The field has recently begun using ecological momentary assessment (EMA) to determine the nature and consequences of abnormalities at each stage of ER. In individuals with SZ, we have found that the identification stage abnormality is best characterized by a threshold that is inefficient, such that ER attempts are too few and conducted with too little effort when the negative effect is high but too frequent and with too much effort when negative affect is low (Raugh & Strauss, 2021; Visser, Esfahlani, Sayama, & Strauss, 2018). At the selection stage, individuals with SZ attempt a wide range of strategies more frequently than healthy controls and engage in more poly-regulation (i.e., attempting multiple strategies simultaneously) (Raugh et al., 2023; Visser, Esfahlani, Sayama, & Strauss, 2018). Furthermore, the strategies individuals with SZ select are less contextually appropriate (Strauss et al., 2019). At the implementation stage, findings consistently indicate that individuals with SZ are less effective at implementing a range of strategies to decrease negative effects in the moment and across time (Kimhy et al., 2020; Li et al., 2023; Ludwig, Mehl, Krkovic, & Lincoln, 2020; Raugh et al., 2023; Raugh & Strauss, 2021; Strauss et al., 2019; Visser, Esfahlani, Sayama, & Strauss, 2018). These implementation stage abnormalities have been found using EMA parallel laboratory-based results, which indicate that individuals with SZ are less effective at decreasing the neurophysiological response to unpleasant stimuli than healthy controls (CN) (Bartolomeo, Culbreth, Ossenfort, & Strauss, 2020; Horan, Hajcak, Wynn, & Green, 2013b; Kim et al., 2021a; Kim et al., 2021b; Morris et al., 2012; Strauss et al., 2013; Strauss et al., 2015; Sullivan & Strauss, 2017; van der Meer et al., 2014; Van Der Velde et al., 2015).

Although past studies suggest that SZ patients display abnormalities at each of the 3 stages of ER that predict clinical outcomes, it is unclear whether the same profile is also observed in CHR (Raugh et al., 2023; Raugh & Strauss, 2021; Strauss et al., 2019; Visser, Esfahlani, Sayama, & Strauss, 2018). The current study used EMA to achieve this aim and evaluate the following hypotheses: (1) CHR will report a greater state negative effect than CN on EMA, consistent with prior studies indicating heightened stress reactivity (Walker et al., 2010; Walker, Mittal, & Tessner, 2008a); (2) At the identification stage, CHR youth will evidence a threshold for regulation that is inefficient (Raugh & Strauss, 2021; Visser, Esfahlani, Sayama, & Strauss, 2018); (3) At the selection stage, CHR youth will select strategies more frequently than CN, engage in greater polyregulation, and select strategies that are less contextually appropriate (Raugh et al., 2023; Strauss et al., 2019; Visser, Esfahlani, Sayama, & Strauss, 2018); (4) At the implementation stage, CHR will be less effective than CN at decreasing the intensity of negative affect from time t to t + 1 (Raugh et al., 2023; Strauss et al., 2019; Visser, Esfahlani, Sayama, & Strauss, 2018); (5) Abnormalities at each ER stage will predict the risk for conversion to a psychotic disorder on the NAPLS risk calculator.

## Methods

### Participants

Participants included 120 CHR youth and 59 CN. CHR participants were recruited from clinical research programs at the University of Georgia, Northwestern University, and Emory University that perform diagnostic evaluations for prodromal psychosis referrals. Community CNs were recruited at the University of Georgia using printed and online advertisements. Study procedures were approved by the local institutional review boards.

The Structured Interview for Psychosis-Risk Syndromes (SIPS) (McGlashan et al., 2001) was used to determine that CHR participants met the criteria for progression or persistence for one or more of the three prodromal syndromes. The SIPS and Structured Clinical Interview for DSM-5 (SCID-5) (First, Williams, Karg, & Spitzer, 2015) were also used to rule out the presence of a lifetime psychotic disorder in CHR. CN did not meet the current diagnostic criteria for any psychiatric disorder on the SCID-5.

CHR and CN did not significantly differ in age, sex, personal education, or parental education (see Table 1); however, there was a lower proportion of Black participants and a greater proportion of White participants among CN compared to CHR. CHR completed significantly fewer EMA surveys than CN (see Table 1). Participants were not excluded based on EMA survey adherence, consistent with methodological recommendations for handling EMA data and the robustness of mixed-effects models in accounting for missing data.

**Table 1.** Participant demographics

Variable	CHR (n = 120, k = 3348)	CN (n = 59, k = 1941)	Test statistic	*p*
Age, M (SD)	22.36 (4.08)	21.47 (3.06)	*F* = 2.17	0.143
Male, n (%)	27 (22.5%)	14 (23.7%)	χ^2^ = 0.03	0.854
Personal education, M (SD)	13.91 (2.69)	14.5 (1.49)	*F* = 2.48	0.118
Parental education, M (SD)	15.31 (2.97)	15.85 (2.83)	*F* = 1.35	0.248
Race, n (%)			χ^2^ = 15.98	0.025
Black/ African American	17 (14.2%)	5 (8.5%)		
Asian American	16 (13.3%)	7 (11.9%)		
Multiracial	12 (10%)	3 (5.1%)		
White	62 (51.7%)	39 (66.1%)		
Hispanic	12 (10%)	4 (6.8%)		
Other	1 (0.8%)	1 (1.7%)		
Adherence, M (SD)	53.22% (28.52%)	71.08% (21.48%)	*F* = 18.05	< 0.001

*Note*. CN = healthy control; CHR = clinical high-risk for psychosis.

### Procedures

Study procedures were completed across three phases.

*Initial Laboratory Visit.* Participants first provided informed consent. Afterward, clinical diagnostic and symptom interviews were performed. Diagnostic and symptom interviews included: the Structured Clinical Interview for DSM-5 (First, Williams, Karg, & Spitzer, 2015), Negative Symptom Inventory Psychosis Risk (Strauss et al., 2023), Global Functioning Scale: Social (Auther, Smith, & Cornblatt, 2006), Global Functioning Scale: Role (Niendam, Bearden, Johnson, & Cannon, 2006). Measures needed for the NAPLS risk calculator score were also administered (Cannon et al., 2016). CHR diagnosis was verified via consensus meetings within and across each site. Next, participants downloaded the mEMA app (ilumivu.com) to their personal smartphone or an Android phone provided for the purposes of the study. Training on app use was provided by research staff and participants completed a practice EMA survey.

*EMA Surveys.* EMA was completed in 6 days. Eight surveys were administered per day across 90 minute epochs from 9 AM to 9 PM. Surveys were available for 15 minutes. On average, surveys took <5 minutes to complete and utilized skip logic to minimize time burden.

EMA items assessed domains of: (1) current emotional experience, (2) emotion regulation, (3) context (location, activity, social companions), and (4) symptoms. Emotional experience items evaluated arousal, negative affect, and positive affect. Positive and negative affect items were based on a modified Differential Emotions scale. Emotion regulation items assessed the decision to regulate (i.e., “Since the last survey, did you try to increase or decrease any of your emotions?”), effort towards regulation, regulatory goal (decrease negative, increase negative, decrease positive, increase positive), strategies employed, emotion regulation self-efficacy, and perceived emotion regulation effectiveness (see Table 2 for item details). Context items evaluated current location (e.g., home, work), activity (e.g., errands, resting), and social interactions (e.g., significant other, friends). Symptom items evaluated the internal experience component of anhedonia, avolition, and asociality, as well as delusions. Context items were also used to generate behavioral indices of avolition (goal-directed activity), anhedonia (recreational activity), and asociality (presence of in-person social interaction with family or friends); these were combined with the internal experience negative symptom items to make composite scores for anhedonia, avolition, and asociality which were then averaged to a single measure of negative symptoms, consistent with procedures for rating these constructs on clinical rating scales that account for behavior and internal experience (Strauss et al., 2023) (see Table 2).

**Table 2.** Ecological momentary assessment items

Construct	Scaling	Item text
Positive affect	0–100^1^	How _____ do you feel right now? Amused, fun-loving, silly Content, serene, peaceful Happy, joyful, glad Love, closeness, trust Proud, confident, self-assured
Negative affect	0–100^1^	How _____ do you feel right now? Angry, irritated, annoyed Sad, downhearted, unhappy Scared, fearful, afraid Ashamed, humiliated, disgraced Anxious, nervous, pressured
ER identification	0 = No, 1 = Yes	Since the last survey, did you try to increase or decrease any of your emotions? *Or* Was there a significant emotional event since the last survey? During the event, did you try to increase or decrease any of your emotions?
ER effort*	0–100^1^	How much effort did you use to try to change your emotion(s) by: shifting your attention? reappraising the situation? hiding them? sharing with others? avoiding the situation?
Anhedonia internal experience	0–100^1^	How much are you enjoying the activity? How much do you think you will enjoy that activity the next time you do it? How much are you enjoying this social interaction? How much do you think you will enjoy interacting with them next time?
Anhedonia behavior	0 = Yes, 1 = No	What are you doing right now? Recreation
Avolition internal experience	0–100	How interested are you in the activity?
Avolition behavior	0 = Yes, 1 = No	What are you doing right now?^2^ Working/studying, errands/ housework, exercising, shopping, or commuting/ traveling
Asociality internal experience	0–100	How interested are you in this social interaction?
Asociality behavior	0 = Yes, 1 = No	Who are you interacting with?^2^ Significant other, family/ roommates, or friends
Negative symptoms	0–200^1^	Anhedonia internal experience × (1 + anhedonia behavior) Avolition internal experience × (1 + avolition behavior) Asociality internal experience × (1 + asociality behavior)
Delusions	0–100^1^	How suspicious do you feel right now? How much is something or someone controlling your thoughts? How much is someone reading your mind or are you reading someone else’s mind?

*Note.* ER = Emotion regulation.

* Only assessed if emotion regulation is endorsed.

1 Average of all items.

2 If any item is selected, 1, otherwise, 0.

*Return to Laboratory.* Participants returned to the lab after the 6-day EMA period to receive compensation in the amount of $30 per hour for interviews and $1 per EMA survey completed. There was also a bonus payment (up to $60) for completing 5 or more surveys per day and > 80% of surveys.

#### Data analysis

All models used multilevel modeling with random intercepts within-person and day to account for nesting and repeated measurements. Random slopes were not used because effects were either based on categorical variables or random slopes resulted in a singular model fit. Multilevel models were selected because they are robust to missing data and thus do not require imputation which could bias time-lagged models. Analyses were conducted using R version 4.2.2.

First, multi-level linear regression was used to examine group differences in state negative emotion reactivity (Hypothesis 1).

Second, two multi-level models examined the nature of abnormalities at the identification (Hypothesis 2) and selection (Hypothesis 3) stages. Multi-level logistic and linear regression examined the effects of group, negative affect, strategy, and the interactions thereof on the rate of emotion regulation attempts and emotion regulation effort, respectively. Significant main effects or interactions involving strategy were followed up by models examining the differential effects of the strategies individually.

Third, the hypothesis of greater poly-regulation in CHR than CN was evaluated using a multilevel linear regression using only instances where regulation was reported (Hypothesis 3).

Fourth, a multilevel linear regression model examined the effects of group, emotion regulation attempt (yes, no), and group x emotion regulation attempt interaction at time t on the change in negative affect from time t to t + 1. Change across time was evaluated through autoregression models (the effect of negative affect at time t on t + 1) (Hypothesis 4).

Fifth, multiple linear regression was used to determine whether abnormalities at each stage predicted risk for conversion on the NAPLS calculator (Hypothesis 5) and whether ER variables added to prediction above and beyond negative emotion reactivity (Hypothesis 6). The following variables were used in the model: (1) negative emotion reactivity (negative affect intensity), identification threshold (mean level of negative affect at which identification was selected), selection (average number of strategies selected), implementation (change in negative affect from time t to t + 1 after making a regulatory attempt), and interactions thereof.

## Results

### Hypothesis 1: negative affect intensity

CHR (M = 21.79, SD = 19.27) reported significantly higher state negative affect than CN (M = 8.55, SD = 11.7) on EMA surveys (χ^2^ = 49.76, p < .001), consistent with increased negative emotion reactivity.

### Hypotheses 2 and 3: identification and selection stages

**Figure 1.**
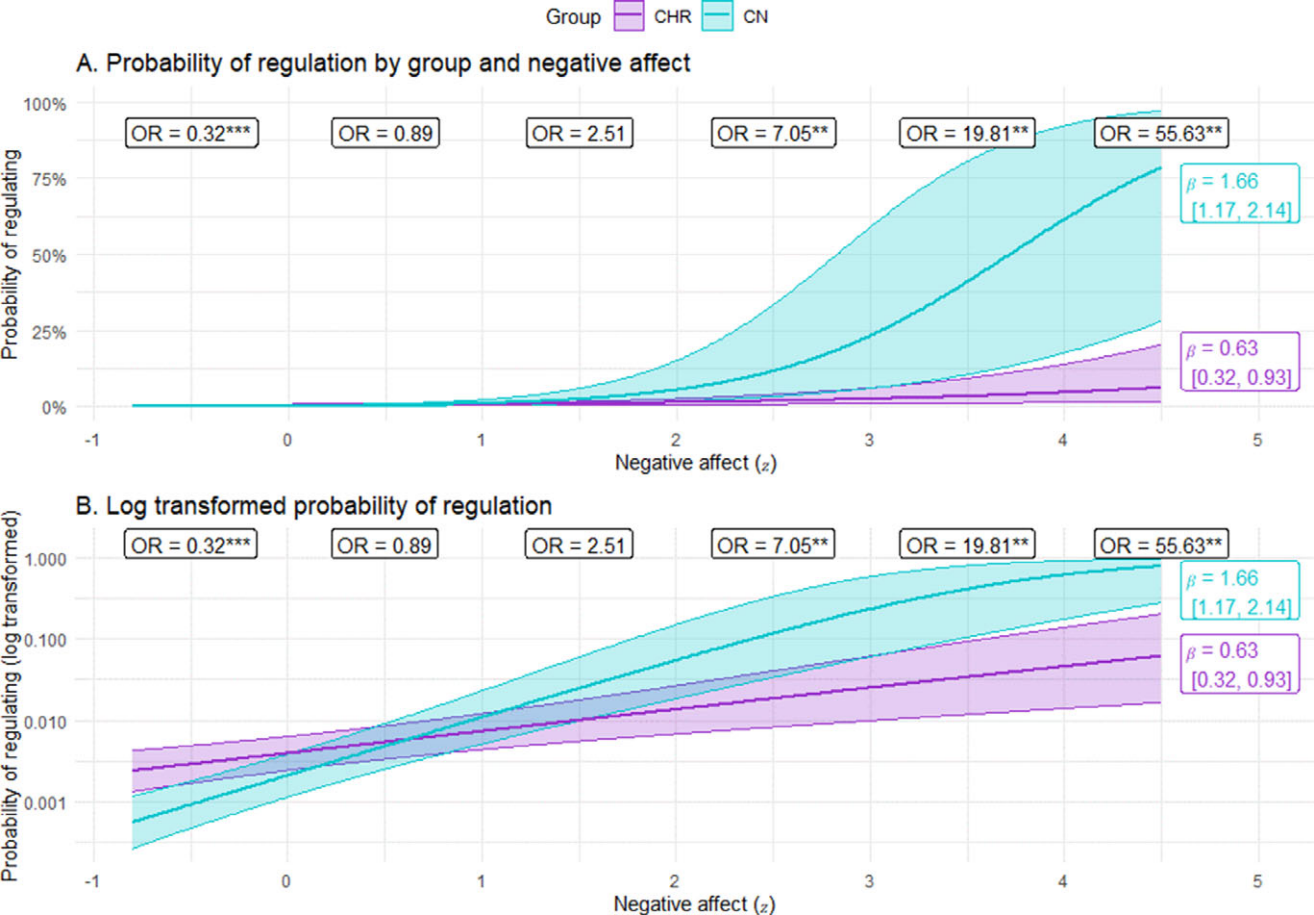
The probability of identifying the need to regulate by group and negative affect. *Note*. CN = healthy control; CHR = clinical high-risk for psychosis; CHR display a threshold for regulation that is inefficient. At high levels of negative affect, CHR regulate less than CN. At low levels of negative affect, CHR regulate more often than CN.

For the rate of attempting to regulate, there was a significant Group x Negative affect interaction, as well as significant main effects for strategy and negative affect. Other main effects and interactions were nonsignificant, although there was a trend toward a significant Group × Negative affect × Strategy interaction (p =0.058) (see Table 3). As depicted in Figure 1, the group x negative affect interaction indicates that CHR are more likely than CN to regulate when negative affect is low but less likely to regulate when negative affect is high. Additionally, the nonsignificant interaction with strategy indicates that this pattern was generally consistent across strategies and thus primarily reflects an identification stage abnormality.

**Table 3.** Identification and selection rate

	Rate		Effort	
	χ^2^	*p*	χ^2^	*p*
Strategy	21.55	<.001	19.98	<0.001
Group	1.25	0.264	0.19	0.661
NA	40.91	<.001	0.67	0.412
Strategy × group	4.22	0.377	11.02	0.026
Strategy × NA	8.57	0.073	12.03	0.017
Group × NA	18.89	<.001	1.39	0.239
Strategy × group × NA	9.15	0.058	13.48	0.009

*Note*. NA = negative affect.

**Figure 2.**
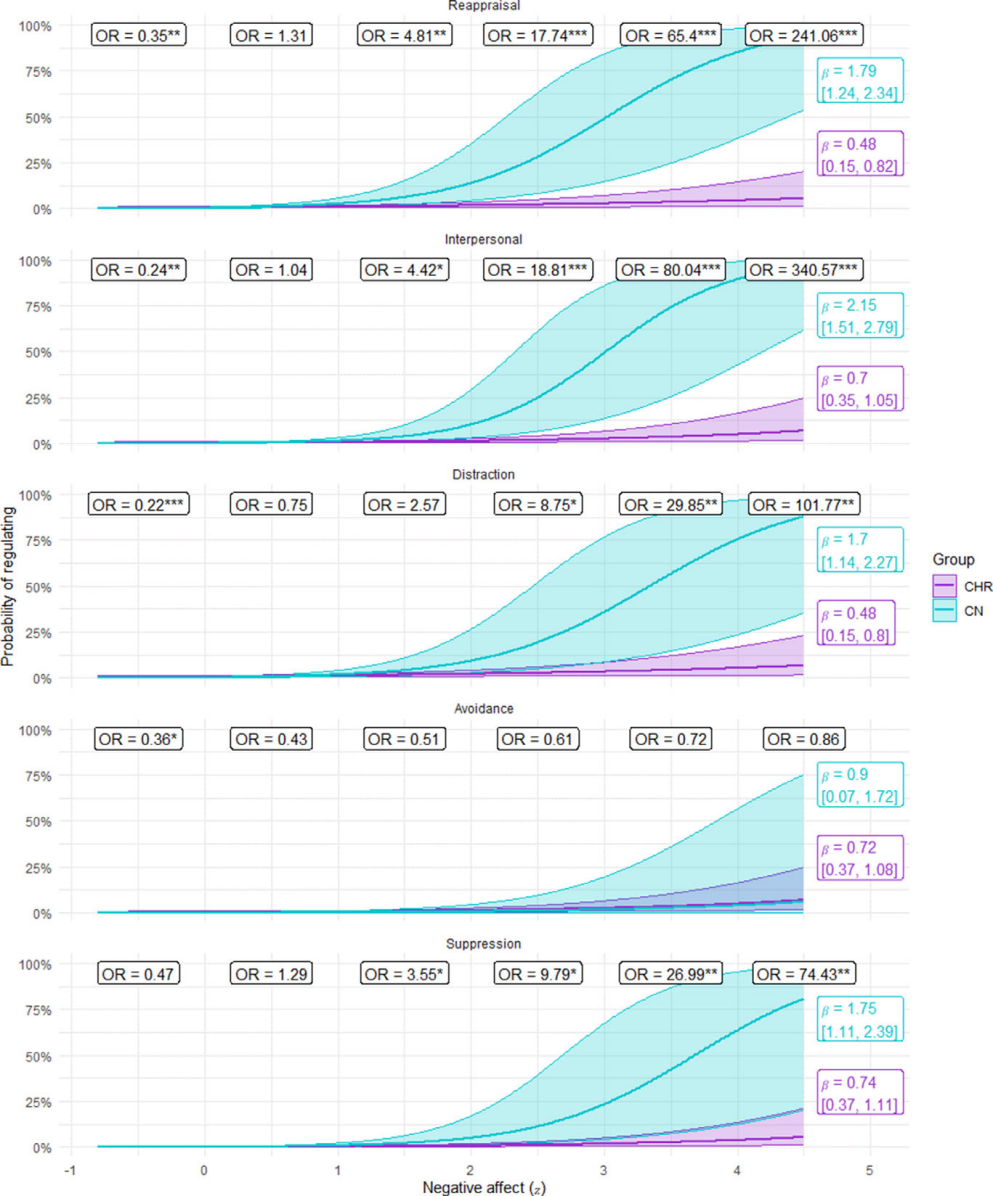
The probability of regulating by group, strategy, and negative affect. *Note*. CN = healthy control; CHR = clinical high-risk for psychosis; At high levels of negative affect, CHR were less likely than CN to select reappraisal, interpersonal, distraction, and suppression strategies. However, groups did not differ in the frequency of selecting avoidance (situation modification) in relation to negative affect levels. At low levels of negative affect, CHR were more likely than CN to select reappraisal, interpersonal, distraction, and avoidance; however, groups did not differ on suppression selection rate when negative affect was low.

However, as depicted in Figure 2, the effect of negative affect was lower for avoidance than the other strategies (i.e., at high levels of negative affect, subjects in both groups were less likely to select avoidance than they were other strategies).

**Figure 3.**
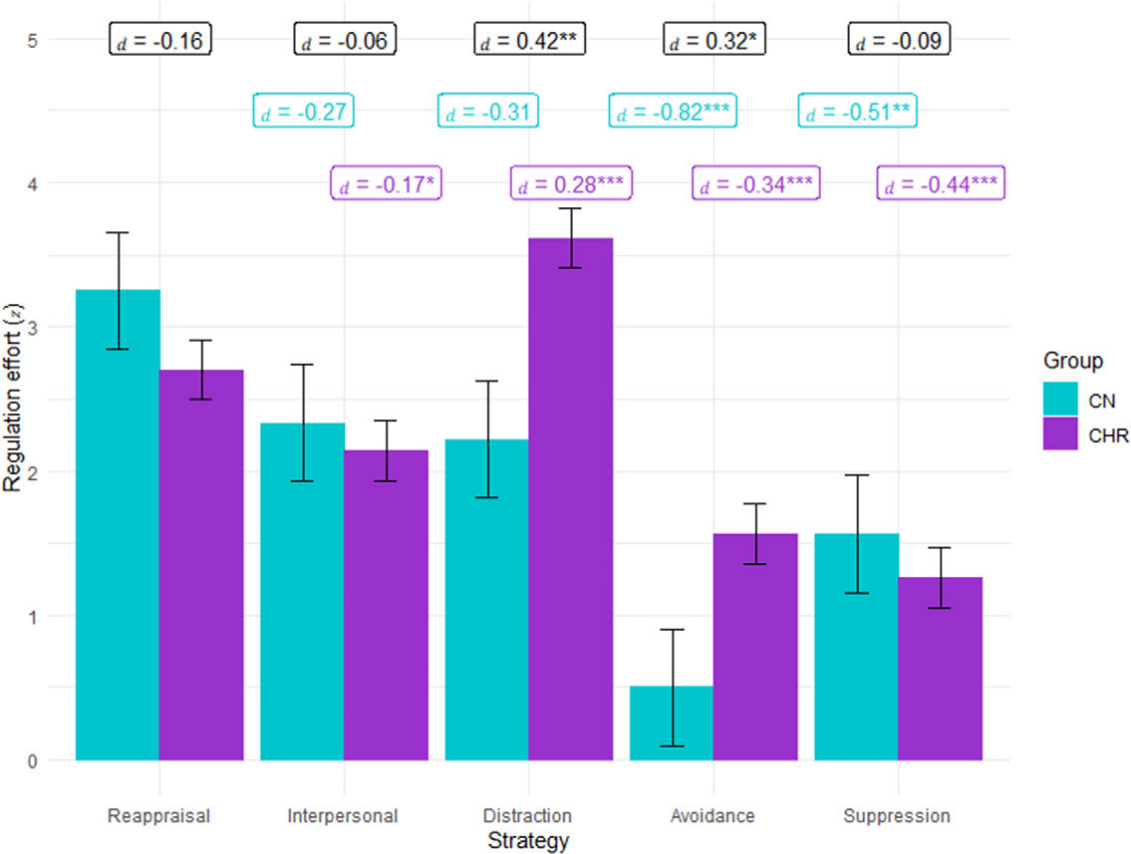
Emotion regulation effort exerted for each group per strategy. *Note*. CHR exerted more effort while regulating than CN when attempting distraction and avoidance; however, groups did not differ for reappraisal, interpersonal, or suppression.

**Figure 4.**
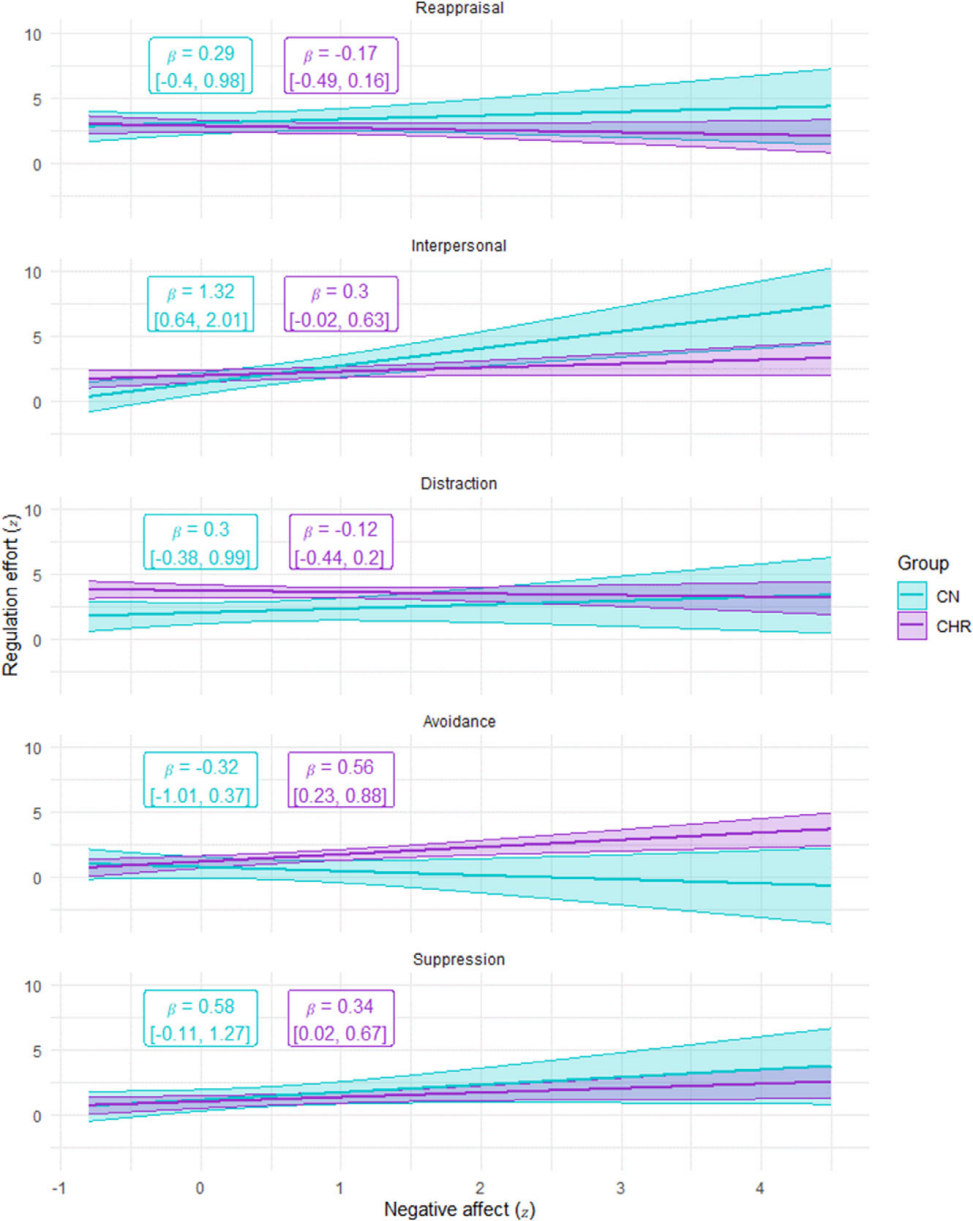
Emotion regulation effort exerted per group based on strategy and level of negative affect. *Note*. CN = healthy control; CHR = clinical high-risk for psychosis.

For emotion regulation effort, there was a significant Group × Negative affect × Strategy three-way interaction, as well as significant two-way interactions of Group × Strategy and Strategy × Negative Affect, and a significant main effect of strategy (see Table 4). As shown in Figure 3, the Group x Strategy interaction indicates that CHR exerted more ER effort than CN for distraction and avoidance, but did not differ on other strategies. However, the three-way interaction presented in Figure 4 clarifies that the level of negative affect differentially impacts how much effort each group exerts when attempting different strategies. At high levels of negative affect, CHR generally exert somewhat less effort than CN; however, this pattern does not hold when attempting avoidance, where CHR exert more effort than CN when negative affect is high. At low levels of negative affect, CHR are more likely to exert higher effort than CN when attempting distraction (also see supplemental materials).

**Table 4.** Predictors of risk for converting to a psychotic disorder on the NAPLS risk calculator in CHR participants

Parameter	Beta	t	p
Trait NA	0.02	0.07	0.946
Reactivity	0.04	0.22	0.830
Identification threshold	−0.03	−0.09	0.932
Distraction	0.10	0.52	0.610
Reappraisal	0.19	1.00	0.326
Suppression	−0.42	−2.36	0.026
Social sharing	−0.13	−0.81	0.424
Avoidance	0.02	0.09	0.927
ER effectiveness	−0.43	−1.8	0.083

*Note*. NA = negative affect.

Regarding polyregulation, CHR were more likely than CN to report attempting multiple strategies at a time: CN: M = 1.59; CHR = 1.90; t = 2.14, p = 0.03; d = 0.26.

### Hypothesis 4: Implementation stage

**Figure 5.**
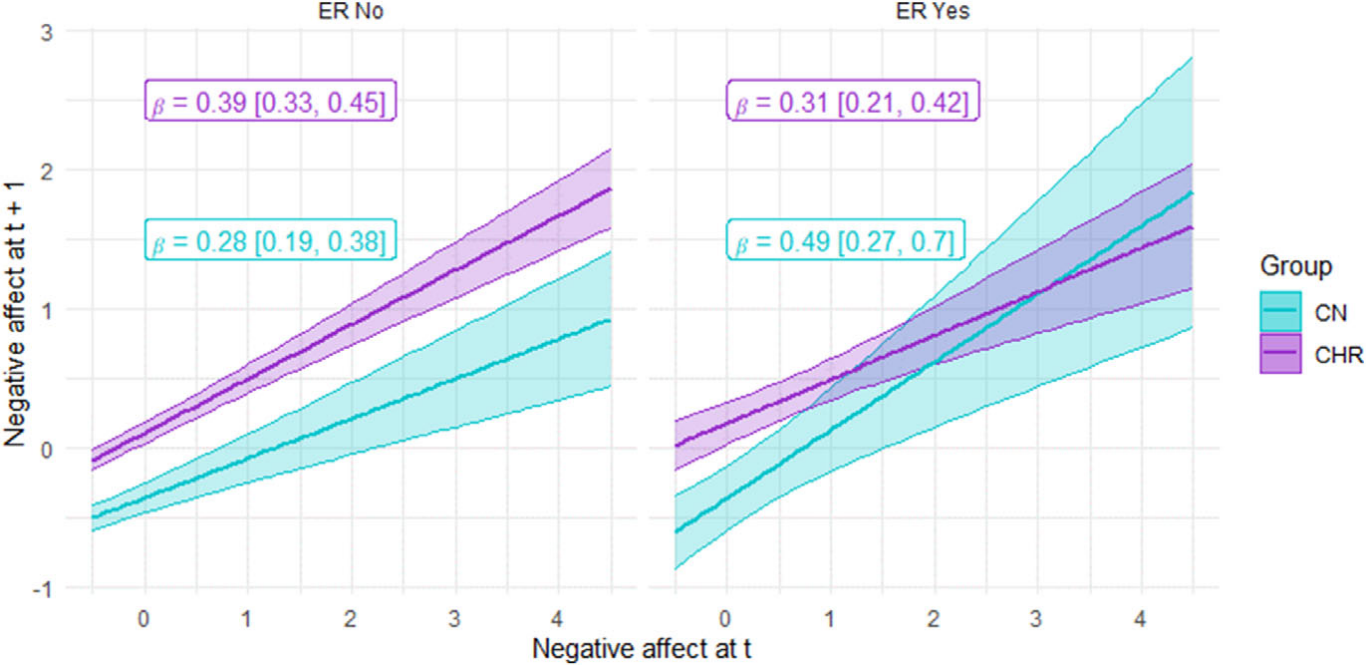
The autoregressive effect of emotion regulation attempts on the change in negative affect across time as an indicator of implementation effectiveness. *Note*. X and Y axis values reflect Z-scores. CN = healthy control. CHR = clinical high-risk for psychosis.

Autoregressive models indicated a significant three-way interaction of Group × Regulation Attempt × Negative affect (χ^2^ = 5.16, *p* = 0.023) and significant main effects of Group (χ^2^ = 49.93, *p* < 0.001) and Negative affect (χ^2^ = 36.87, *p* < 0.001); the effects of Regulation (χ^2^ = 0, *p* = 0.991), Group × Regulation (χ^2^ = 0.28, *p* = 0.599), Negative affect × regulation (χ^2^ = 3.39, *p* = 0.065), and Group X Negative affect (χ^2^ = 3.63, *p* = 0.057) were nonsignificant. As depicted in Figure 5, when there is no regulatory attempt, NA increases over time in both groups. Consistent with increased reactivity when not making ER attempts, NA is higher at time t in CHR than CN and remains higher at t + 1, increasing similarly in both groups. In contrast, groups display a different pattern when there is a regulatory attempt. As depicted in the bottom left quadrant of Figure 5, when NA is lower at time t and CN regulate, their attempt is more effective than CHR at reducing NA at t + 1. However, both groups are relatively equally ineffective at reducing NA via ER attempts when NA is high at t (top right quadrant of Figure 5). The observation that the autoregressive slope does not change in CHR suggests their ER attempts are equally ineffective when NA at time t is low or high. However, ER attempts are more effective when NA is low than high at time t in CN. Thus, ER is generally easier when NA is low than high for most people, but for CHR, ER attempts are ineffective regardless of NA level.

### Hypotheses 5 and 6: ER stage predictors of conversion risk

Greater frequency of selecting suppression was a significant predictor of risk for conversion to psychosis on the NAPLS risk calculator and there was a trend toward poor implementation being significant (see Table 4). However, no effects survived correction for multiple comparisons.

**Figure 6.**
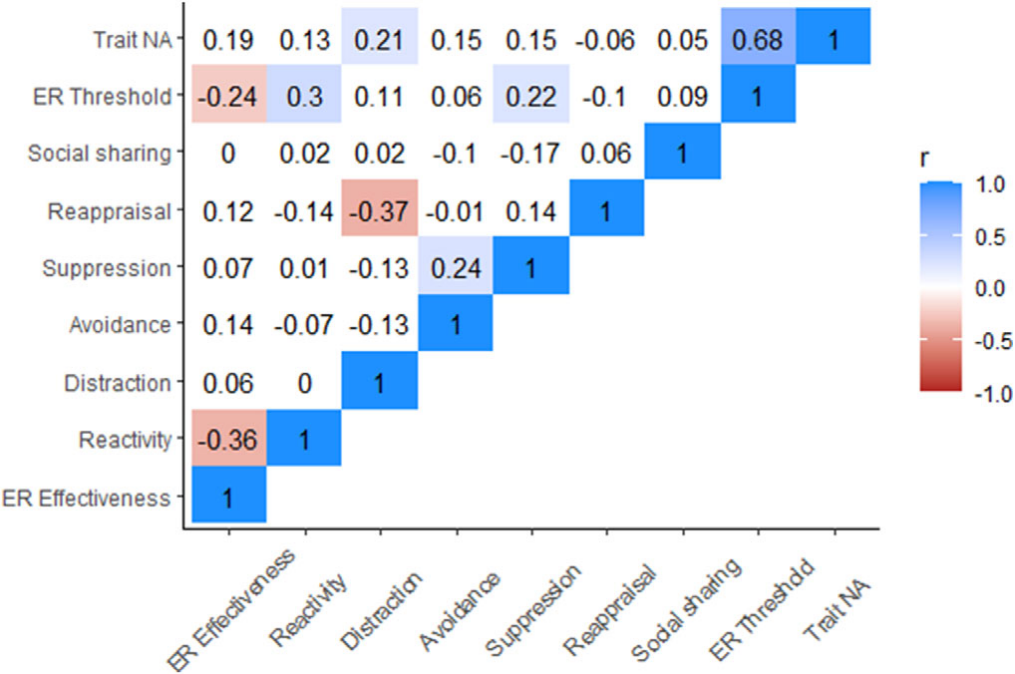
Correlations among the three stages of emotion regulation. *Note*. Cells without a colored background are nonsignificant.

Exploratory analyses:

Exploratory correlations were conducted to examine associations among the 3 stages of ER. Results indicated that greater trait negative affect was associated with greater emotion regulation threshold and use of distraction; greater emotion regulation threshold was associated with lower emotion regulation effectiveness, greater emotional reactivity, and increased use of suppression; cognitive reappraisal was negatively associated with distraction; suppression was positively correlated with avoidance; and greater emotional reactivity was associated with reduced emotion regulation effectiveness (see Figure 6).

## Discussion

The current study examined the nature of emotion regulation abnormalities in participants at CHR for psychosis in relation to the three stages of Gross’ extended process model and whether these abnormalities predicted risk for conversion. Findings are discussed in relation to each stage.

### Identification

Consistent with hypotheses, CHR made regulatory attempts more often than CN and with too much effort at low levels of negative effect. When the negative affect was high, CHR made fewer regulatory attempts than CN and exerted insufficient effort. These findings parallel what has been observed in outpatients with SZ using these same EMA methods (Raugh & Strauss, 2021) and suggest that the threshold for regulation is best characterized as inefficient. Like people with SZ (Raugh & Strauss, 2021; Visser, Esfahlani, Sayama, & Strauss, 2018), CHR participants have difficulty determining the emotional contexts in which it is most adaptive to make ER attempts. The identification stage may serve as an early bottle-neck that feeds forward to produce problems with subsequent ER stages. Identification has three substeps that unfold sequentially: perception, valuation, and action (Gross, 2015). Dysfunction within these sub-steps may lead to inaccuracies in determining when it is most adaptive to identify the need for regulation (Sheppes, Suri, & Gross, 2015). The inefficient identification threshold observed in CHR likely results from a combination of abnormalities at the three sub-steps. Heightened perception and over-valuation of down-regulating negative affective states may contribute to excessive regulation at low levels of negative affect, whereas low emotion regulation efficacy and poor emotion regulation knowledge may reduce the probability of initiating ER attempts when negative affect is high (Macfie et al., 2023).

### Selection

At the selection stage, CHR evidenced greater polyregulation and increased effort expenditure when attempting distraction and avoidance. These findings are consistent with hypotheses and prior EMA studies examining outpatients with SZ, which indicated increased effort and greater polyregulation compared to CN (Raugh et al., 2023; Visser, Esfahlani, Sayama, & Strauss, 2018). Additionally, greater frequency of selecting suppression predicted increased risk for conversion on the NAPLS risk calculator, consistent with prior trait questionnaire findings in CHR and SZ indicating that suppression in particular is associated with poor clinical outcomes (Chapman et al., 2020; Horan, Hajcak, Wynn, & Green, 2013a; Kimhy et al., 2012; Kimhy et al., 2016; Livingstone, Harper, & Gillanders, 2009; Perry, Henry, & Grisham, 2011; Rowland et al., 2013; van der Meer, van’t, & Aleman, 2009).

Why might individuals at CHR and those with SZ be more likely to use more than one ER strategy at a time to decrease negative affect in daily life? In healthy individuals, greater intensity of NA results in higher rates of polyregulation (Barrett, Gross, Christensen, & Benvenuto, 2001). High rates of NA are common in CHR and may drive more frequent polyregulation as a functional compensatory mechanism (i.e., a single strategy may not be complex enough to be effective so multiple strategies are attempted) (Strauss et al., 2019). Similarly, increased rates of emotional co-activation (i.e., simultaneously experiencing negative and positive emotions) have been associated with elevated polyregulation (Ford, Gross, & Gruber, 2019). Since increased emotional co-activation has been observed in SZ (Sanchez, Lavaysse, Starr, & Gard, 2014; Strauss, Visser, Lee, & Gold, 2017), it is possible that higher rates of polyregulation occur because emotional co-activation drives CHR to select a multitude of goals/strategies/tactics to regulate the concurrently experienced negative and positive emotions. Alternatively, CHR participants may differ in the extent to which they use contextual information to guide their use of emotion regulation strategies (Raugh et al., 2023). They may be less able to represent relevant features of a context and use those representations to guide strategy selection and implementation, potentially due to reduced fund of emotion regulation knowledge (Strauss, Visser, Lee, & Gold, 2017). Engaging in more contextually insensitive polyregulation likely results in regulatory attempts that are less functional and effective (i.e., implementation stage abnormalities). CHR and SZ may also experience negative emotions as more inconsistent with their ideal affective state than non-clinical peers (James, Berglund, Chang, & Strauss, 2022), thus motivating a greater frequency and intensity of efforts to regulate unwanted negative emotions.

### Implementation

At the implementation stage, there was support for an impairment in down-regulating negative affect following ER attempts in CHR. Specifically, auto-regression indicated that at low levels of negative affect at time t, CN were more effective at using ER to decrease negative affect at t + 1 than CHR. However, both groups were equally ineffective at decreasing negative affect from t to t + 1 when negative affect was high. Additionally, the autoregressive slope did not differ in CHR, suggesting that they were equally ineffective at decreasing negative affect across time regardless of whether it was low or high at time t, whereas CN was more effective at implementing strategies when negative affect was low than high. Abnormalities at the implementation stage were at a trend level of significance predicting risk for conversion to a psychotic disorder on the NAPLS risk calculator. Thus, implementation stage abnormalities may also be of clinical relevance.

Evidence for impaired implementation obtained here via EMA is consistent with prior studies indicating that individuals at CHR are less effective than CN at down-regulating neurophysiological responses to unpleasant laboratory-based stimuli (Kim et al., 2021a). When considered in conjunction with findings obtained from similar paradigms in SZ and prior EMA results that evidence similar effects (Bartolomeo, Culbreth, Ossenfort, & Strauss, 2020; Horan, Hajcak, Wynn, & Green, 2013b; Kim et al., 2021a; Kim et al., 2021b; Kimhy et al., 2020; Li et al., 2023; Ludwig, Mehl, Krkovic, & Lincoln, 2020; Morris et al., 2012; Raugh et al., 2023; Raugh & Strauss, 2021; Strauss et al., 2013; Strauss et al., 2015; Strauss et al., 2019; Sullivan & Strauss, 2017; van der Meer et al., 2014; Van Der Velde et al., 2015; Visser, Esfahlani, Sayama, & Strauss, 2018), findings suggest that implementation stage abnormalities occur across phases of psychosis.

### Associations among the three stages of ER

Exploratory analyses indicated that abnormalities at the identification stage (higher threshold) are related to a greater likelihood of selecting suppression (a maladaptive strategy) and poorer effectiveness at down-regulating negative affect (implementation stage). Thus, abnormalities in the identification stage may have downstream effects on subsequent stages and identification may be a critical treatment target.

### Limitations

Certain limitations should be considered. First, the study was cross-sectional, and conclusions regarding the impact of ER on conversion cannot be drawn. The NAPLS risk calculator that was calculated using the cross-sectional data provides some suggestion that selection and implementation stage deficits may be particularly relevant for conversion; however, this cannot be confirmed without longitudinal data. Second, statistical power was limited by the frequency of ER attempts across the sample. Future studies may benefit from collecting EMA data over a longer timeframe with more samples. Third, the extended process model was used to select strategies and guide interpretation. This assumes that ER attempts are explicit and focus on the use of specific strategies. However, other models and modes (e.g., implicit) of ER exist, other strategies can be explored that were not considered, and alternate stages can be considered. Future studies may therefore benefit from taking a multimodal approach that incorporates additional strategies and conceptual approaches.

## Conclusions

Findings suggest that the nature of ER abnormalities at the identification, selection, and implementation stages is highly similar between CHR and SZ. At the identification stage, abnormalities are best characterized as an inefficient regulatory threshold (i.e., attempts were made too frequently at low negative affect and not frequently enough at negative affect). At the selection stage, abnormalities are characterized by greater polyregulation. Implementation stage abnormalities reflect being less effective at decreasing the intensity of negative affect following an ER attempt. The abnormalities observed across the three ER stages may reflect dysfunctional interactions between emotional salience processing and cognition. For example, dysfunctional emotion-attention interactions may result in more rapid automatic processing of unpleasant stimuli, along with greater difficulty shifting attention while implementing strategies. Impairments in valuation may contribute to over or undervaluing certain strategies in certain contexts, and difficulty updating valuation judgments based on experience due to working memory deficits. Abnormalities in effort-cost computation may also influence how often, at which intensity, and how long individuals engage in ER attempts. Future studies are needed to identify common and distinct mechanisms underlying the three ER stages, particularly using laboratory-based paradigms.

Although heightened stress reactivity is widely considered an important mechanism underlying the development of psychosis among those at CHR, there is emerging evidence suggesting that ER abnormalities may also contribute above and beyond stress response. The consistency of abnormalities observed across phases of illness suggests that there may be common ER treatment targets that can be pursued. Psychosocial treatments, such as emotion regulation therapy, have been developed and proven effective in internalizing disorders that share some common ER abnormalities (Mennin & Fresco, 2014). Such interventions could be adapted for use in SZ-spectrum populations where they may hold similar potential for improving negative affect and symptoms.

## Supporting information

Strauss et al. supplementary material Strauss et al. supplementary material

## Supplementary material

The supplementary material for this article can be found at http://doi.org/10.1017/S0033291724003155.
