# Supplementary material for: Deconstructing the nature of emotion regulation impairments at the identification, selection, and implementation stages in individuals at clinical high-risk for psychosis
Source: Psychol Med. 2025 Feb 5;55:e22. doi: 10.1017/S0033291724003155 (PMC12017363; doi:10.1017/S0033291724003155)

**SUPPLEMENTAL MATERIALS**

**DECONSTRUCTING THE NATURE OF EMOTION REGULATION IMPAIRMENTS AT THE IDENTIFICATION, SELECTION, AND IMPLEMENTATION STAGES IN INDIVIDUALS AT CLINICAL HIGH-RISK FOR PSYCHOSIS**

Gregory P. Strauss, Ph.D. ^1^*

Ian M. Raugh, M.S. ^1^

Katherine F. Visser, Ph.D. ^2^

Elaine F. Walker, Ph.D. ^3^

Vijay A. Mittal, Ph.D. ^4^

1. Department of Psychology, University of Georgia
2. Department of Psychiatry, Alpert School of Medicine, Brown University
3. Department of Psychology, Emory University
4. Department of Psychology, Northwestern University

Correspondence concerning this article should be addressed to Gregory P. Strauss, Ph.D., Email: gstrauss@uga.edu. Phone: +1-706-542-0307. Fax: +1-706-542-3275. University of Georgia, Department of Psychology, 125 Baldwin St., Athens, GA 30602.

**Supplemental Analyses:**

Covariate Analysis 1:

Analyses were reconducted using ethnicity as a covariate. The pattern of results was highly similar and there was no indication that ethnicity had an important impact on emotion regulation.

Table S1. Models Recalculated using Ethnicity as a Covariate

|  | Rate | |
| --- | --- | --- |
|  | *χ^2^* | *p* |
| Group | 3.36 | 0.067 |
| NA | 62.65 | < .001 |
| Ethnicity (White vs not-White) | 0.92 | 0.338 |
| Group X NA | 23.27 | < .001 |

Covariate Analysis 2:

The pattern of results was similar when sex and education were entered as coavriates. Neither sex nor education had significant effects on emotion regulation variables

Table S2. Models Recalculated using Sex and Education as Covariates

|  | Rate | |
| --- | --- | --- |
|  | *χ^2^* | *p* |
| Group | 2.74 | 0.098 |
| NA | 63.22 | < .001 |
| Sex | 3.89 | 0.048 |
| Group X NA | 23.86 | < .001 |
|  | Rate | |
|  | *χ^2^* | *p* |
| Group | 3.3 | 0.069 |
| NA | 60.4 | < .001 |
| Personal education | 0.73 | 0.39 |
| Group X NA | 23.61 | < .001 |

Exploratory Correlations:

Exploratory correlations examined associations between EMA ER variables averaged at the 1 week level and summary level clinical data from rating scales. Results indicated no significant associations between emotion regulation variables and symptom interview measures.


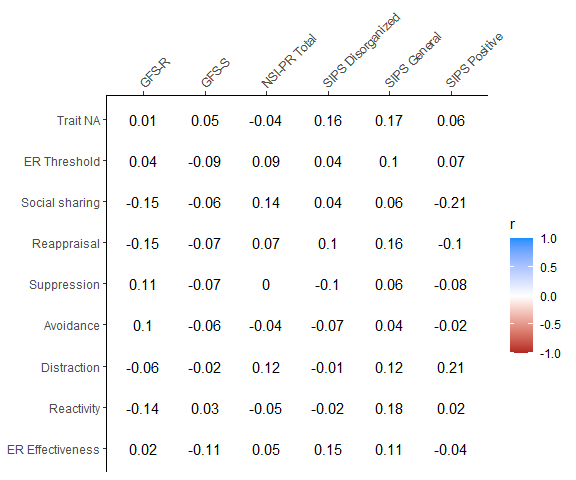


Exploratory Analyses Examining Context:

Analyses examining context had too few EMA survey instances to be reliably interpreted:

|  | Rate | |
| --- | --- | --- |
|  | *χ^2^* | *p* |
| Group | 2.89 | 0.089 |
| NA | 62.94 | < .001 |
| Home context | 0.08 | 0.895 |
| Group X NA | 23.17 | < .001 |
|  | Rate | |
|  | *χ^2^* | *p* |
| Group | 2.89 | 0.089 |
| NA | 63.58 | < .001 |
| Social context | 0.7 | 0.404 |
| Group X NA | 22.76 | < .001 |
|  | Rate | |
|  | *χ^2^* | *p* |
| Group | 2.95 | 0.086 |
| NA | 62.34 | < .001 |
| Goal-directed context | 0.14 | 0.708 |
| Group X NA | 23.19 | < .001 |

Figure S1. Log Transformed Version of Figure 2 in the manuscript to assist with effect visualization.


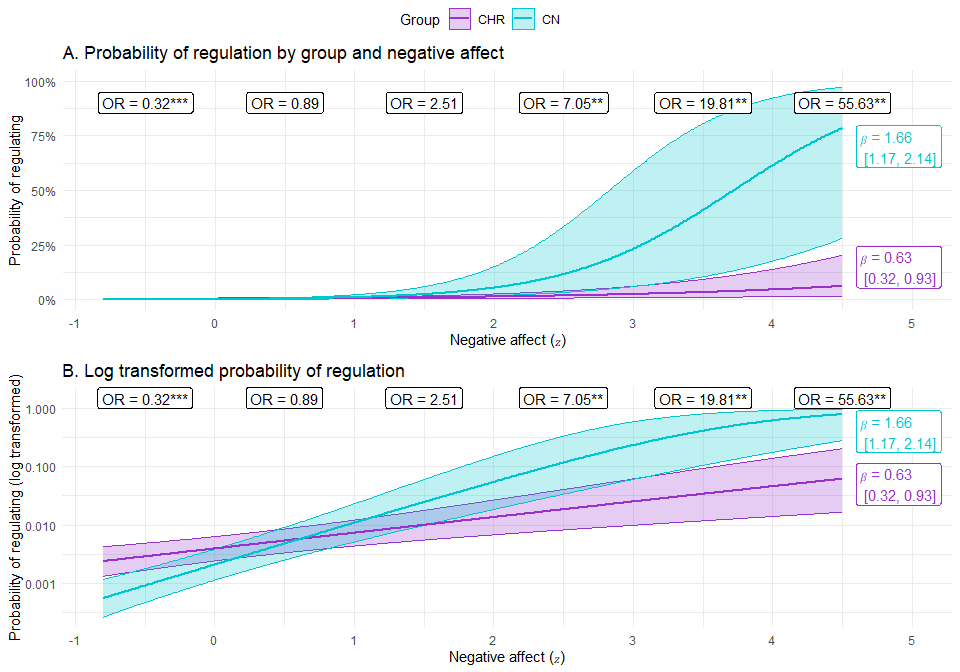


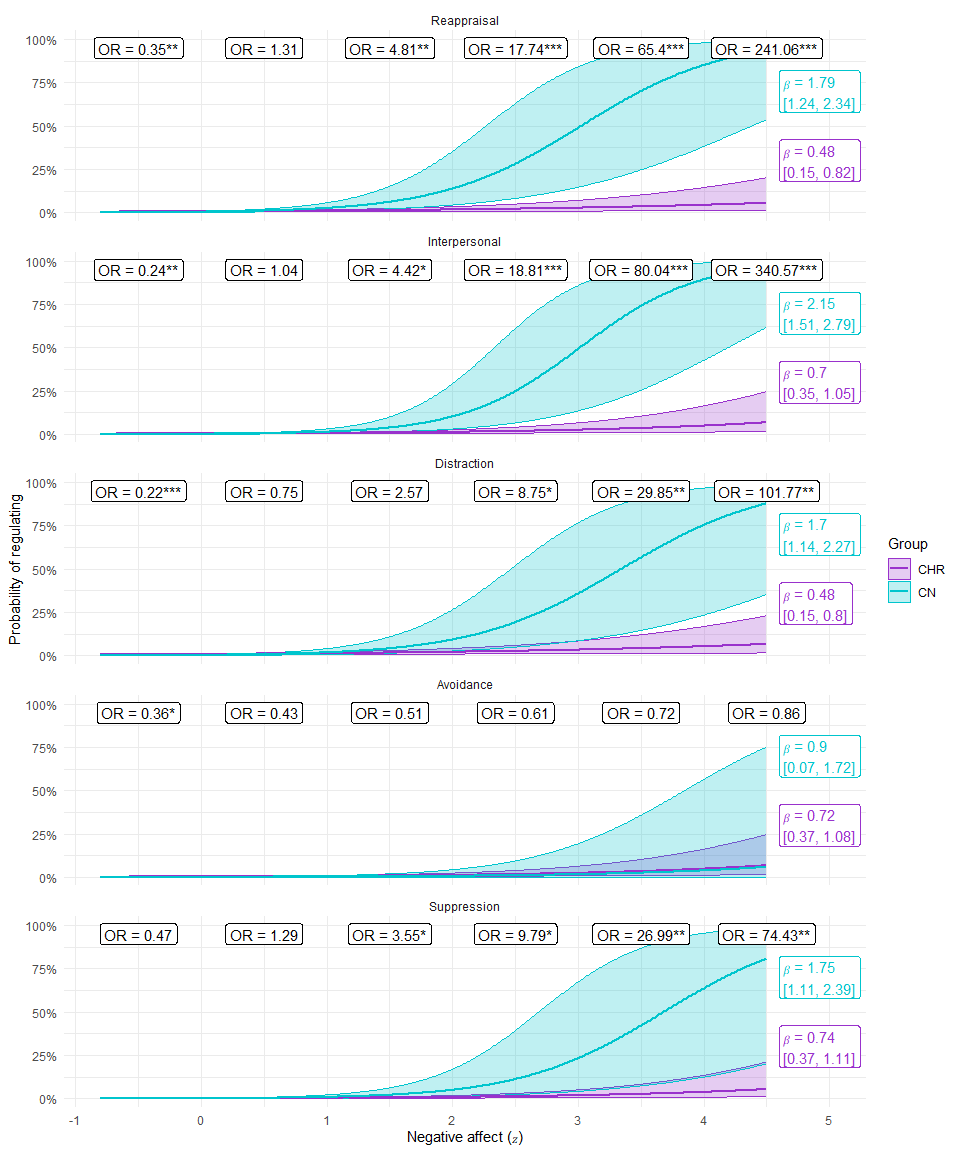


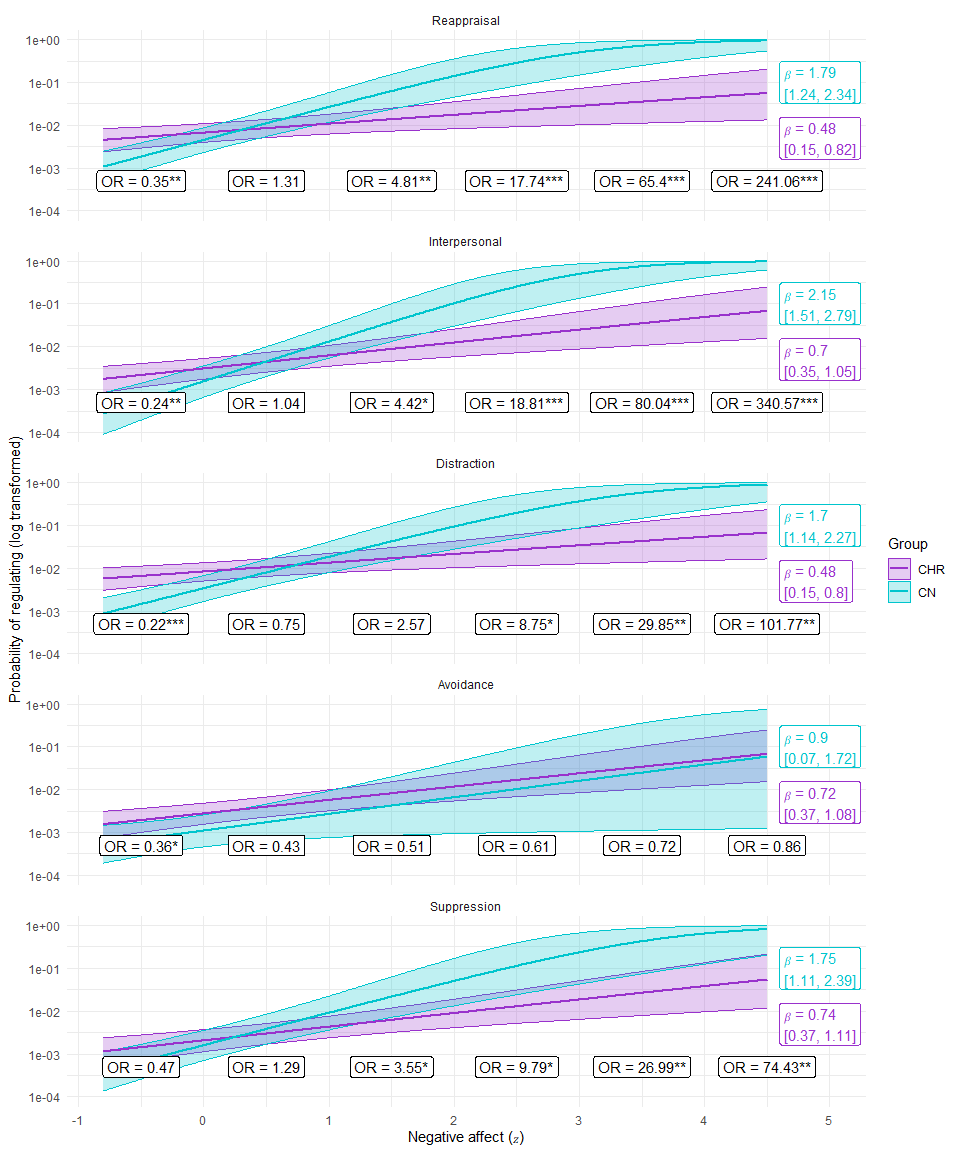

Supplement: Strauss et al. supplementary material [file S0033291724003155sup001.docx]
